# Xanthomatous Discoid Lupus Erythematosus: An Unusual Histopathological Variant and Review of Reported Cases

**DOI:** 10.7759/cureus.96509

**Published:** 2025-11-10

**Authors:** Suthasanee Prasertsook, Waranya Boonchai, Penvadee Pattanaprichakul

**Affiliations:** 1 Department of Dermatology, Faculty of Medicine, Siriraj Hospital, Mahidol University, Bangkok, THA; 2 Department of Pathology, Faculty of Medicine, Siriraj Hospital, Mahidol University, Bangkok, THA

**Keywords:** discoid lupus erythematosus (dle), foam cells, histopathological varirants, lipid-laden macrophage, xanthomatous change

## Abstract

Discoid lupus erythematosus (DLE) is the most common form of cutaneous lupus erythematosus and may lead to chronic scarring. Xanthomatous changes, characterized by dermal lipid-laden macrophages, are exceedingly rare, with only three cases reported to date. We present a patient with classic DLE lesions whose biopsy demonstrated CD68-positive foam cells and granular deposition of IgM, IgA, and C3 along the dermo-epidermal junction. Systemic evaluation revealed no abnormalities. The patient was treated with topical and systemic corticosteroids, resulting in partial clinical improvement. This fourth reported case underscores the importance of clinicopathologic correlation and further expands the recognized histopathological spectrum of DLE.

## Introduction

Discoid lupus erythematosus (DLE) is the most prevalent subtype of cutaneous lupus erythematosus, typically presenting with well-demarcated erythematous to violaceous plaques, follicular plugging, and a propensity for permanent scarring and pigmentary alterations. Diagnosis is often clinical but may require histopathological confirmation, particularly in atypical or chronic lesions [1,2]. Characteristic histopathologic features include vacuolar interface dermatitis, perivascular and periadnexal lymphocytic infiltration, basement membrane thickening, and dermal mucin deposition. The occurrence of dermal lipid-laden macrophages, or xanthomatous changes, is extremely uncommon in DLE, with only three cases reported worldwide [3,4]. These xanthomatous features can closely resemble xanthomas, granulomatous dermatoses, or lymphoproliferative disorders, highlighting the importance of clinicopathologic correlation. Herein, we describe the fourth reported case of xanthomatous DLE, emphasizing its rarity and the clinical relevance of recognizing this distinctive histopathological variant.

## Case presentation

**Figure 1:**
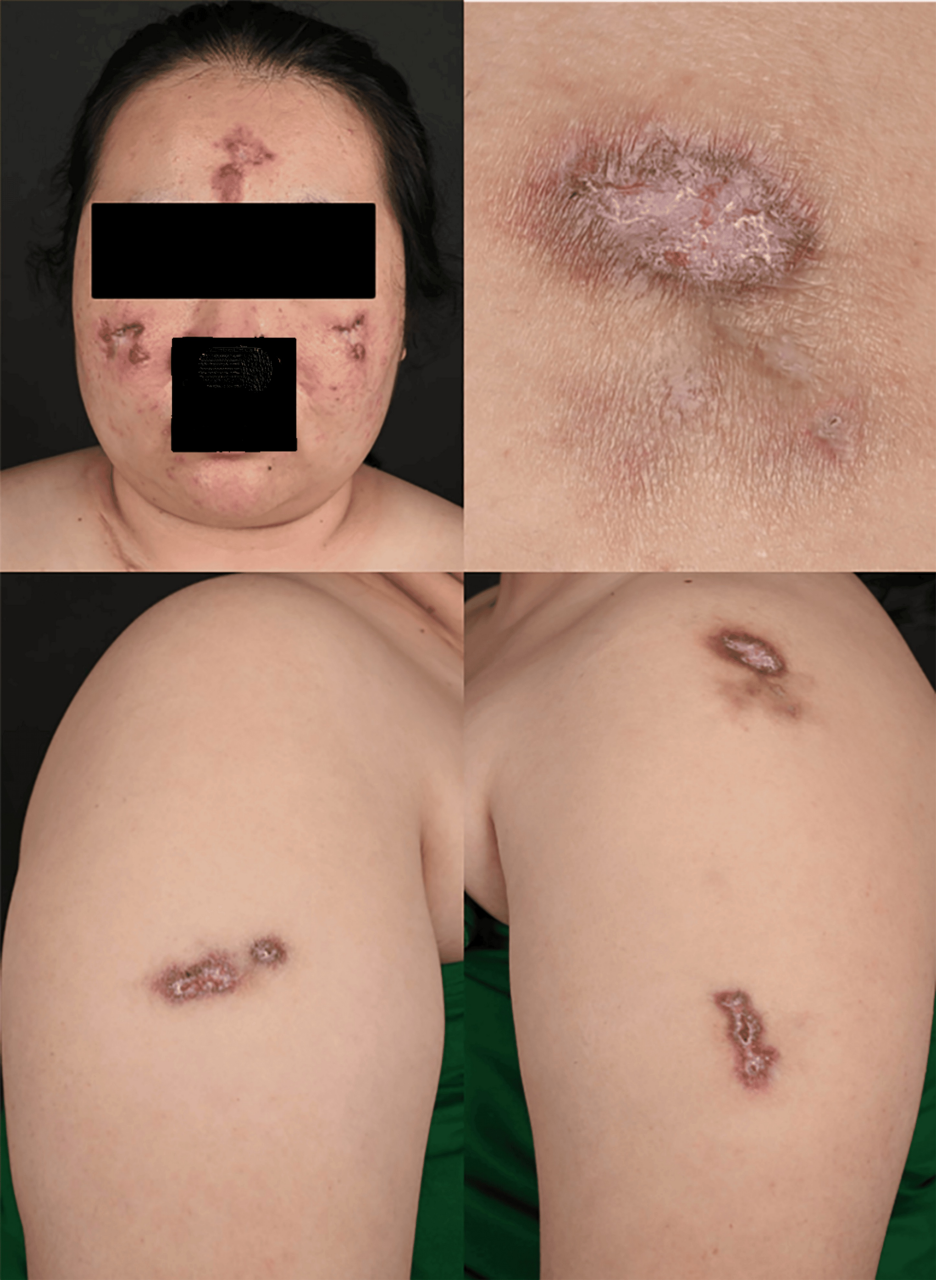
Clinical presentation: Multiple discrete, well-demarcated asymptomatic atrophic violaceous plaques with a brownish rim and central atrophy on the upper arms, forehead, and cheeks.

A 28-year-old female presented with a 10-year history of slowly progressive, asymptomatic atrophic plaques on her upper arms, forehead, and cheeks. A physical examination revealed multiple discrete, well-demarcated violaceous plaques with a brownish rim and central atrophy (Figure 1). She denied any history of trauma, rash, oral ulcer, alopecia, arthralgia, or other systemic symptoms. No similar skin lesions were reported in her family. 

**Figure 2:**
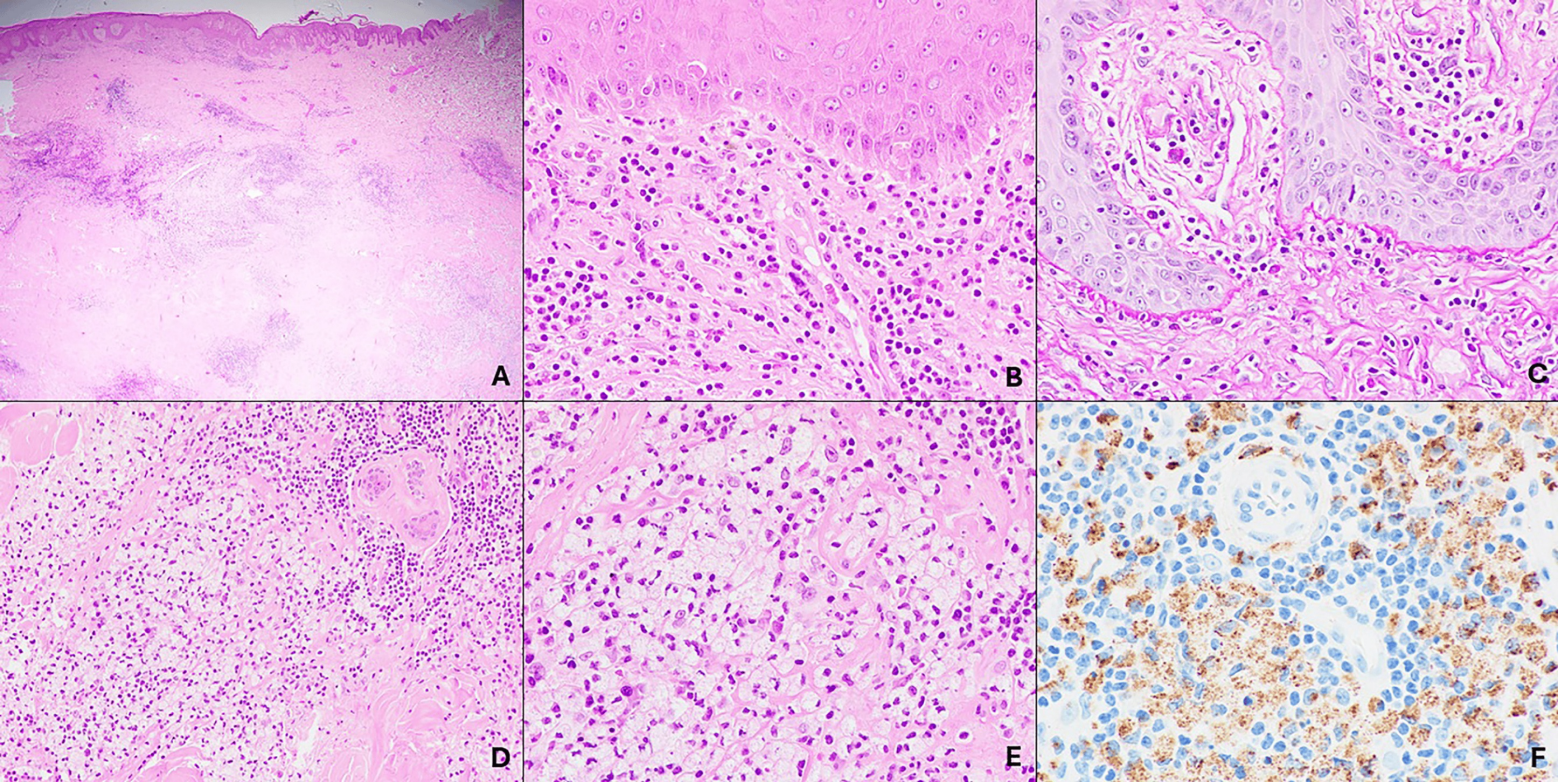
Microscopic and immunohistochemical features. Microscopic examination revealed an acanthotic epidermis with compact orthokeratosis, basal vacuolization, and scattered necrotic keratinocytes with superficial and deep perivascular and peri-adnexal lymphocytic infiltrates (A, B; H&E x20 and x400 magnification, respectively). The periodic acid-Schiff (PAS) stain highlighted focally thickened basement membrane (C; PAS x400 magnification), and numerous lipid-laden histocytes with lymphoplasmacytic infiltrates in the dermis (D, E; H&E x200 and x400 magnification, respectively). CD68 immunohistochemistry showed positivity among the lipid-laden histocytes (F; CD68 x400 magnification).

**Figure 3:**
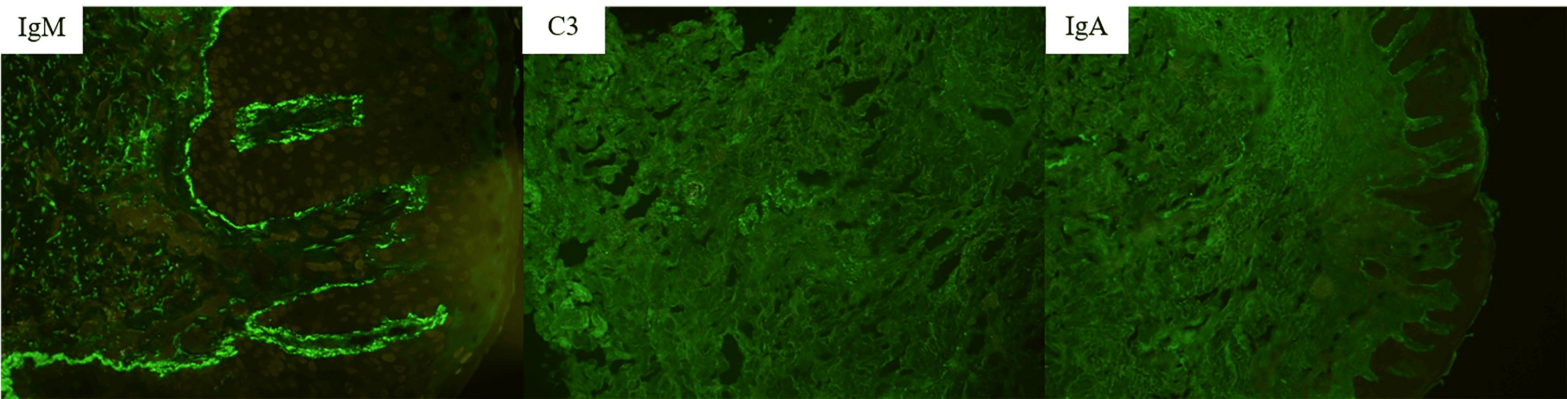
Direct immunofluorescent study. Direct immunofluorescence demonstrated positivity for immunoglobulin M (intensity 3+), C3 (intensity 1+), and immunoglobulin A (intensity 1+) in a granular pattern at the dermo-epidermal junction and around the appendages.

Recently, she developed a well-defined erythematous plaque with an adherent scale on her left upper arm. Based on clinical suspicion of discoid lupus erythematosus, an incisional biopsy was performed at the lesion on the left shoulder. Histopathologic examination showed a hyperkeratotic epidermis with basal vacuolization and scattered necrotic keratinocytes. There were prominent superficial and deep perivascular and peri-adnexal lymphocytic infiltrate with some plasma cells, a thickened basement membrane, and lipid-laden histocytes with lymphoplasmacytic infiltrate in the interstitial dermis. CD68 immunostaining was positive among the lipid-laden histocytes (Figure 2). Direct immunofluorescence (DIF) revealed immunoglobulin M (intensity 3+), C3 (intensity 1+), and immunoglobulin A (intensity 1+) in a granular pattern along the dermo-epidermal junction and appendages, suggesting an immune complex-mediated process (Figure 3). Based on these findings, a diagnosis of xanthomatous discoid lupus erythematosus was established. 

The patient’s complete blood count, renal function tests, lipid profile, stool examination, complement levels (C3 and C4), and immunologic investigations, including antinuclear antibody (ANA), anti-double-stranded DNA (anti-dsDNA), anticardiolipin antibodies, anti-β2 glycoprotein I antibodies, and lupus anticoagulant, were all within normal limits. Liver function tests demonstrated mildly elevated transaminase levels, with otherwise normal hepatic parameters. Further evaluation revealed mild hepatic steatosis on imaging study, and hepatitis screening (HBsAg and anti-HCV antibody) was negative. A summary of the laboratory findings is presented in Table 1. An ophthalmologic examination was unremarkable. Consequently, oral hydroxychloroquine was initiated at a dose of 400 mg/day, in combination with intralesional and topical corticosteroids. However, the patient later developed a mild morbilliform drug eruption without systemic involvement, which was attributed to hydroxychloroquine hypersensitivity, leading to discontinuation of the medication. Subsequently, oral prednisolone at 10 mg/day was prescribed for DLE management. Systemic treatment with oral corticosteroid was slowly tapered and stopped after four months, since no additional lesions emerged and the previous lesions demonstrated sufficient improvement. At the eight-month follow-up, atrophic scarring from previous lesions was noted, along with the development of new DLE lesions in the malar region. These were treated with once-daily topical mometasone furoate, which resulted in only minimal improvement.

**Table 1:** Summary of the patient’s laboratory investigation. Hb, hemoglobin; Hct, hematocrit; WBC, white blood cell count; BUN, blood urea nitrogen; Cr, creatinine; AST, aspartate aminotransferase; ALT, alanine aminotransferase; ALP, alkaline phosphatase; TB, total bilirubin; DB, direct bilirubin; HDL, high-density lipoprotein cholesterol; LDL, low-density lipoprotein cholesterol; ANA, antinuclear antibody; anti-dsDNA, anti-double-stranded DNA

Test item		Patient’s results	Reference range	Units
Complete blood count	Hb	13.3	12.0-14.9	g/dL
	Hct	41.2	37.0-45.7	%
	WBC	7.10	4.4-110.3	×10⁹/L
	Platelets	295	179-435	×10⁹/L
Renal function tests	BUN	13.7	6.0-20.0	mg/dL
	Cr	0.58	0.51-0.95	mg/dL
Liver function tests	AST	59	8-40	U/L
	ALT	64	7-56	U/L
	ALP	68	35-104	U/L
	TB/DB	0.30/0.17	0.0-1.2/0.0-0.3	mg/dL
	Albumin/Globulin	4.2/3.5	3.5-5.2/1.5-3.5	g/dL
Lipid profile	CHOL	170	<200	mg/dL
	TG	113	<150	mg/dL
	HDL	60	<60	mg/dL
	LDL	87	<100	mg/dL
Stool examination	Microscopy/Occult blood	Negative	Negative	-
Immunology	Complement C3, C4	149, 31	83-177, 15-45	mg/dL
	ANA	Negative	Negative	-
	Anti-dsDNA	Negative	Negative	-
	Anticardiolipin IgG	<2	<4	GPL.U/mL
	Anticardiolipin IgM	<2	<12	MPL.U/mL
	Anti-Beta-2-GP1 IgG	<2	<20	RU/mL
	Anti-Beta-2-GP1 IgM	2	<20	RU/mL
	Lupus anticoagulant	Negative	Negative	-

## Discussion

DLE, the most prevalent form of cutaneous lupus erythematosus, typically begins as a well-demarcated, coin-shaped erythematous to violaceous lesion with adherent scale and follicular plugging. These lesions may progress to permanent atrophic scarring, pigmentary alterations, and disfigurement [1]. DLE presents in two clinical forms: a localized form (approximately 80% of cases), where lesions are confined to sun-exposed areas above the neck, and a disseminated form (20%), in which lesions extend below the neck and are associated with a higher risk of progression to systemic lupus erythematosus (SLE) [1,2]. Histologically, DLE is characterized by vacuolar dermatitis, superficial and deep perivascular and peri-adnexal lymphohistiocytic infiltration, basement membrane thickening, and dermal mucin deposition. Follicular plugging is also common in older lesions [1].

We report a case of clinically classic cutaneous DLE with rare histopathologic findings, notably the presence of prominent xanthomatized macrophages within the dermis. The accumulation of lipid-laden foam cells is an uncommon phenomenon, seen in both hyperlipidemic and normolipidemic individuals. The presence of xanthomatized macrophages in cutaneous lupus erythematosus is exceedingly rare, with only four prior cases previously documented worldwide: three in DLE [3,4], and one in lupus panniculitis [5]. Table 2 summarizes the clinical characteristics, histology, and comorbidities of these reported cases [3,4]. In all cases, including ours, xanthomatous features were observed histologically, although only one case exhibited clinical features resembling xanthomas. Furthermore, only one case was diagnosed concurrently with SLE [3-5]. 

**Table 2:** Clinical characteristics, histopathological features, and comorbidities of reported cases of xanthomatous DLE. DLP, dyslipidemia; SLE, systemic lupus erythematosus; N/A, not available; LDL, low-density lipoprotein

Authors (year of publication)	Sex, Age (years)	Morphology	Histology	SLE	DLP
Netherton (1945) [3]	Female, 45	-Localized form: face, and ear; gradual yellowing of plaques	Few dilated hair follicles orifices with hyperkeratotic plugs, dermal infiltrate with lymphocytes, fibroblasts, epithelioid cells, and foam cells varied in sizes, few atrophic sebaceous glands	N/A	Absent
Weiss et al. (2020) [4]	Male, 34	-Generalized form: scalp, face, torso, and arms; no xanthomatous appearance	Vacuolar degeneration, thickened basement membrane, perivascular and perifollicular lymphohistiocytic infiltration, collection of lipidized histiocytes in dermis (CD68 +, CD1a -)	Yes	Absent
	Female, 43	-Localized form: scalp; no xanthomatous appearance	Vacuolar degeneration, thickened basement membrane, perivascular and periadnexal lymphohistiocytic infiltration, increased dermal mucin, fibrotic tracts at sites of extinct hair follicles, collection of lipid-laden histiocytes in dermis	No	Present (Mildly elevated cholesterol and LDL)
This study	Female, 28	-Generalized form: face, and arms; no xanthomatous appearance	Vacuolar degeneration and scattered necrotic keratinocytes, perivascular and periadnexal inflammatory infiltration, thickened basement membrane, lipid-laden histocytes with lymphoplasmacytic infiltrate in the dermis (CD68 +)	No	Absent

The accumulation of xanthomatized macrophages has been documented in a variety of disorders, including noninflammatory diseases, inflammatory diseases, and neoplastic cutaneous diseases, such as mycosis fungoides and other lymphoproliferative disorders. Most of these cases present clinically as dystrophic xanthomatosis, which contrasts with previously reported cases of cutaneous lupus erythematosus [4].

The pathogenesis of xanthomatized macrophage accumulation remains poorly understood. Some authors have suggested that lipid material released from epithelial damage and keratinocyte degeneration or from neoplastic cell destruction following therapy in lymphoproliferative disorders is phagocytized by dermal macrophages, leading to the formation of foam cells and xanthomatous lesions. Another condition characterized by a dermal xanthomatous infiltrate is verruciform xanthoma (VX), which has been reported in association with various chronic dermatoses, including DLE [6]. Evidence from light and electron microscopic studies of VX suggests that trauma and chronic inflammation can induce VX via the mechanism mentioned above. An alternative hypothesis proposes that leakage of lipoproteins from small blood vessels, followed by their uptake by dermal cells, may also contribute to foam cell accumulation [4]. Therefore, similar mechanisms may contribute to the xanthomatous changes observed in our case of DLE.

## Conclusions

Xanthomatous discoid lupus erythematosus is an exceptionally rare histological variant, with only four cases reported to date. The presence of dermal lipid-laden macrophages can closely mimic xanthoma, granulomatous dermatoses, or lymphoproliferative disorders, highlighting the need for careful clinicopathological correlation to avoid misdiagnosis. Reporting this case not only expands the recognized histological spectrum of cutaneous lupus erythematosus but also provides insight into possible mechanisms of foam cell formation in chronic inflammatory skin diseases. 

## References

[1] Elman SA, Joyce C, Nyberg F (2017). Development of classification criteria for discoid lupus erythematosus: Results of a Delphi exercise. J Am Acad Dermatol.

[2] Chong BF, Song J, Olsen NJ (2012). Determining risk factors for developing systemic lupus erythematosus in patients with discoid lupus erythematosus. Br J Dermatol.

[3] Netherton EW (1945). Chronic discoid lupus erythematosus with superimposed xanthomatous infiltration: report of a case. Arch Dermatol Syphilol.

[4] Weiss A, Kwon EJ, Kreidel M, Mann R, Cohen JA, Hui Y, Jacobson MI (2020). Two unusual cases of discoid lupus erythematosus associated with xanthomatized macrophages. Am J Dermatopathol.

[5] Ishikawa O, Akimoto S, Sato M, Okugi Y, Takeuchi Y, Miyachi Y (1996). Xanthomatous reaction in lupus panniculitis. Acta Derm Venereol.

[6] Meyers DC, Woosley JT, Reddick RL (1992). Verruciform xanthoma in association with discoid lupus erythematosus. J Cutan Pathol.

